# DNA Methylation Signatures Associated With Smoking Status Across Lung Cancer Subtypes Using The Cancer Genome Atlas (TCGA) Methylation Data

**DOI:** 10.7759/cureus.106555

**Published:** 2026-04-06

**Authors:** Blessing T Ojinna, Abimbola E Arisoyin, Akinyele Oladimeji, Chigozirim Okpechi, Azeberoje Osueni

**Affiliations:** 1 Internal Medicine, Guthrie Lourdes Hospital, Binghamton, USA; 2 Global Health, Emory University Rollins School of Public Health, Atlanta, USA; 3 Psychiatry, Harlem Hospital Center, New York, USA; 4 Family Medicine, Obafemi Awolowo University, Ile-Ife, NGA; 5 Medicine, St George’s University, St. George's, GRD; 6 Gastroenterology and Hepatology, Lagos University Teaching Hospital, Lagos, NGA

**Keywords:** dna methylation, epigenetics, lung cancer, non-small cell lung cancer, smoking, tcga

## Abstract

Background: Lung cancer remains the leading cause of cancer-related mortality worldwide, with cigarette smoking as its strongest etiologic factor. Beyond genetic mutations, smoking induces epigenetic alterations, particularly DNA methylation, which may differ across lung cancer subtypes and contribute to tumor heterogeneity.

Objective: The objective of this study was to identify smoking-associated differentially methylated CpG sites (FDR < 0.05) across lung cancer subtypes using The Cancer Genome Atlas (TCGA) 450K methylation data, and to enhance biological interpretation by annotating their genomic context and functional relevance, including associated genes and regulatory features.

Methodology: This retrospective observational study analyzed Illumina HumanMethylation450 BeadChip data from TCGA lung adenocarcinoma (LUAD) and lung squamous cell carcinoma (LUSC) cohorts. Multivariable linear models within the limma framework assessed differential methylation between smokers and never smokers, adjusting for age, sex, tumor stage, and histology. False discovery rate (FDR) correction was applied.

Results: A total of 771 patients (92 never smokers, 679 smokers) were analyzed. Seven CpG sites were significantly associated with smoking status (FDR < 0.05), with absolute methylation differences (Δβ) ranging from 0.100 to 0.123. Subtype-specific analyses identified eight LUAD-specific and five LUSC-specific smoking-associated CpG sites. LUAD exhibited both hypermethylation and hypomethylation patterns, whereas LUSC showed predominantly smoking-related hypomethylation.

Conclusion: Smoking is associated with distinct and subtype-specific DNA methylation signatures in lung cancer, underscoring its role in epigenetic tumor modulation and supporting the potential utility of methylation markers in lung cancer stratification and risk assessment.

## Introduction

Worldwide, lung cancer has remained the leading cause of cancer mortality and has been found to constitute a significant proportion of cancer deaths in the world [1]. Regardless of the progress in diagnosis and treatment, prognosis is poor in most patients with the disease, mainly because the diagnosis is late, and the disease is biologically heterogeneous [2]. The most important contribution to the etiology of lung cancer is cigarette smoking, which causes most, but not all, cases, and molecular and genetic factors play an essential role in lung carcinogenesis [3,4]. The role of smoking in promoting the molecular changes in various subtypes of lung cancer is thus important in enhancing risk stratification, early diagnosis, and treatment decisions [5].

Lung cancer can be categorized into two major types: non-small cell lung cancer (NSCLC) and small cell lung cancer (SCLC), with around 85% of the overall cases comprising NSCLC [6]. NSCLC is a heterogeneous disease that is characterized by a number of histological subtypes, two of which, lung adenocarcinoma (LUAD) and lung squamous cell carcinoma (LUSC), are the most common and clinically significant ones [7]. Both LUAD and LUSC belong to the NSCLC category, but they differ significantly when it comes to cellular origin, molecular features, and the correlation with smoking exposure [8]. LUSC, similar to SCLC, is highly linked to cigarette smoking, usually occurs in the central airways, and has a high mutational burden, which is caused by carcinogens associated with tobacco [7]. In comparison, LUAD is more commonly found in never-smokers and ex-smokers; it is more common in the peripheral lung areas, and the dissimilar oncogenic driver changes are predominantly epidermal growth factor receptor (EGFR) mutations and anaplastic lymphoma kinase (ALK) rearrangements, which bear significant therapeutic significance [9]. These variations demonstrate that LUAD and LUSC, although classified under NSCLC, are biologically different diseases with different patterns of pathogenesis in relation to smoking, which is noticeably differentiated in relation to SCLC and demonstrates the necessity of molecular and epigenetic studies of the subtypes [7].

In addition to genetic mutations, epigenetic modifications, especially of DNA methylation, are very important in smoking-induced lung carcinogenesis [10]. DNA methylation is the process of attaching methyl groups to the cytosine base, which usually results in transcriptional repression in cases where the changes take place in the gene promoters [11]. Tobacco smoke is rich in carcinogens that have the potential of causing expansive epigenetic imbalances, which lead to aberrant methylation patterns that lead to malignant alteration [12].

Smoking-related DNA methylation signatures have already become the prospective risk assessment, subtype classification, and prognosis biomarkers of lung cancer [13]. These methylation marks might be cumulative smoking exposures and could vary in different subtypes of lung cancer, and provide information on subtype-specific pathogenesis [14]. Moreover, alterations in methylation can have a direct effect on gene expression, which leads to pathways of cell cycle control, DNA repair, inflammation, and immune response [15]. The connection between smoking-related patterns of methylation and gene expression is thus needed in order to clarify functional implications and find biologically applicable targets [16].To improve biological interpretability, CpG sites can be contextualized within their genomic features and linked to nearby genes and regulatory elements. Annotation of differentially methylated CpG loci (e.g., promoter regions, gene bodies, and CpG island contexts) enables functional insight into potential roles in tumor-related pathways, including immune response, DNA repair, and cellular proliferation. Incorporating such annotations is essential for translating epigenetic findings into biologically meaningful and clinically relevant insights.

Clinically, the epigenetic changes have a lot of therapeutic and diagnostic implications. In comparison to genetic mutations, DNA methylation modifications are potentially reversible and thus are a desirable target of epigenetic therapies, including DNA methyltransferase inhibitors [17]. Besides, it is also possible to identify methylation markers in minimally invasive samples such as blood and sputum, which allows identifying lung cancer at an early stage and following the progression of the disease, especially in high-risk smokers [18]. Alam et al. demonstrated systemic effects of smoking on oral and respiratory tissues, reinforcing the need to consider exposure‑driven molecular changes across anatomical sites [19].

The study utilizes The Cancer Genome Atlas (TCGA) methylation data for the analysis. TCGA represents an extensive publicly accessible resource of multi-omics, which includes the DNA methylation and gene expression profiles of the various subtypes of lung cancer [20]. Utilizing TCGA methylation information can be used to conduct a large-scale exploration of epigenetic modifications in smoking-related phenomena in an established clinical and molecular context [21]. This study aims to identify smoking-associated differentially methylated CpG sites across lung cancer subtypes and evaluate subtype-specific epigenetic patterns using TCGA methylation data.

## Materials and methods

Study design and data source

This was a retrospective, observational study utilizing publicly available data from TCGA. DNA methylation and corresponding clinical data for lung cancer were obtained from the TCGA-LUAD and TCGA-LUSC projects through the Genomic Data Commons (GDC) data portal [22,23]. DNA methylation data were obtained using the GDC portal via the TCGAbiolinks R package. The following query parameters were used: project = c("TCGA-LUAD", "TCGA-LUSC"), data category = "DNA Methylation", data type = "Methylation Beta Value", and platform = "Illumina HumanMethylation450". Data were downloaded using the GDC API method. It was then processed as beta values, representing the proportion of methylation at individual CpG sites. Clinical annotations were downloaded from TCGA and harmonized with molecular data using unique TCGA sample identifiers.

Study population

The study population consisted of patients with primary lung cancer enrolled in the TCGA-LUAD and TCGA-LUSC cohorts who had available DNA methylation data and complete smoking status information. Samples were included only if they had corresponding clinical data and passed quality control steps during data alignment. Patients with missing smoking status or incomplete sample identifiers that prevented reliable matching between clinical and methylation data were excluded. The final analytical cohort comprised patients with lung adenocarcinoma or lung squamous cell carcinoma with complete and aligned molecular and clinical information.

Study variables and measures

The primary exposure variable was smoking status, categorized as smoker or never smoker for the main analyses to improve interpretability and statistical stability. Tumor subtype was defined based on TCGA project designation (TCGA-LUAD or TCGA-LUSC). Additional covariates included age at diagnosis (measured in years), sex, and pathological tumor stage. Pathological stage categories were harmonized from original American Joint Committee on Cancer (AJCC) stage annotations and grouped into stages I-IV for analysis.

The primary outcome was DNA methylation level at individual CpG sites, measured as beta values ranging from 0 (unmethylated) to 1 (fully methylated). Differential methylation was assessed at the CpG level. CpG sites were annotated to nearby genes using Illumina HumanMethylation450 annotation data to facilitate biological interpretation.

Statistical analysis

All statistical analyses were conducted using R software version 4.5 (R Foundation for Statistical Computing, Vienna, Austria, https://www.R-project.org/). Descriptive statistics were used to summarize baseline clinical and demographic characteristics of the study population. Continuous variables were reported as means with standard deviations, while categorical variables were summarized as frequencies and percentages. Group differences between smoking status categories were assessed using independent samples t-tests for continuous variables and chi-square tests for categorical variables, as appropriate. These comparisons were used to generate the baseline characteristics table.

Differential DNA methylation associated with smoking status was assessed using multivariable linear models implemented in the limma framework. For each CpG site, methylation beta values were modeled as the dependent variable, with smoking status as the primary independent variable, adjusting for age at diagnosis, sex, pathological tumor stage, and histological subtype. Empirical Bayes moderation was applied to stabilize variance estimates across CpG sites and improve statistical power. P-values were adjusted for multiple testing using the Benjamini-Hochberg false discovery rate (FDR) method.

Subtype-specific analyses were performed by fitting separate multivariable models within the LUAD and LUSC cohorts using the same covariate structure. Differentially methylated CpG sites were considered statistically significant at an FDR < 0.05, with effect sizes examined to prioritize biologically meaningful methylation differences.

A post hoc power assessment indicated that, given the sample size (92 never smokers and 679 smokers) and observed effect sizes (Δβ ≈ 0.10), the study had adequate statistical power (>80%) to detect meaningful differences in methylation levels at individual CpG sites under typical variability assumptions.

Handling of missing data

Samples with missing smoking status or incomplete clinical identifiers were excluded prior to analysis. For the remaining covariates, analyses were restricted to complete cases to ensure consistency across multivariable models. CpG sites with missing methylation values across samples were removed before model fitting. No imputation was performed for missing molecular or clinical data.

Ethical considerations

This study used de-identified, publicly available data from TCGA. As all data were previously collected and released in compliance with ethical standards and informed consent procedures, institutional review board approval and additional patient consent were not required for this secondary analysis.

## Results

Table 1 presents the baseline demographic and clinicopathologic characteristics of lung cancer patients stratified by smoking status (never smokers versus smokers).

**Table 1:** Baseline demographic and clinicopathologic characteristics of TCGA lung cancer patients by smoking status Continuous variables are presented as mean ± standard deviation and compared using independent samples t-tests. Categorical variables are presented as number (percentage) and compared using chi-square (χ²) tests. Smokers included current and former smokers. Tumor subtype was defined based on The Cancer Genome Atlas (TCGA) project classification into lung adenocarcinoma (TCGA-LUAD) and lung squamous cell carcinoma (TCGA-LUSC). Pathologic stage was classified according to the American Joint Committee on Cancer (AJCC) staging system.* *p < 0.05 was considered statistically significant. Statistically significant p-values are indicated with an asterisk (*)

Variable	Never smokers (n=92)	Smokers (n=679)	test	p-value
Diagnosis age (years), mean±SD	65.49±10.03	66.81±9.54	t = -1.19	0.215
Gender, n(%)	–	–	χ2 = 11.73	0.001*
Male	38 (41.3%)	412 (60.7%)	–	–
Female	54 (58.7%)	267 (39.3%)	–	–
Subtype, n(%)	–	–	χ2 = 16.97	<0.001*
TCGA-LUAD	70 (76.1%)	358 (52.7%)	–	–
TCGA-LUSC	22 (23.9%)	321 (47.3%)	–	–
AJCC Pathologic stage, n(%)	–	–	χ2 = 4.70	0.195
I	39 (42.4%)	358 (52.7%)	–	–
II	30 (32.6%)	197 (29.0%)	–	–
III	18 (19.6%)	105 (15.5%)	–	–
IV	5 (5.4%)	19 (2.85)	–	–

A total of 771 lung cancer patients were included in the analysis, comprising 92 never smokers and 679 smokers. The mean age at diagnosis was comparable between the two groups, with never smokers having a mean age of 65.49 ± 10.03 years and smokers having a mean age of 66.81 ± 9.54 years. This difference was not statistically significant (t = −1.19, p = 0.215), indicating that age distribution at diagnosis was similar across smoking categories.

Sex distribution differed significantly by smoking status (χ² = 11.73, p = 0.001). Among never smokers, females constituted the majority, accounting for 54 (58.7%) individuals, while males accounted for 38 (41.3%) individuals. In contrast, smokers were predominantly male, with 412 (60.7%) males compared to 267 (39.3%) females. This pattern highlights a strong association between smoking status and sex within this lung cancer cohort.

Tumor subtype also varied significantly between never smokers and smokers (χ² = 16.97, p < 0.001). LUAD was more prevalent among never smokers, observed in 70 (76.1%) patients, whereas smokers showed a more balanced distribution between subtypes, with 358 (52.7%) cases classified as TCGA-LUAD and 321 (47.3%) cases classified as TCGA-LUSC. This finding aligns with known epidemiologic differences in lung cancer subtypes by smoking exposure.

In contrast, no statistically significant difference was observed in AJCC pathologic stage distribution between the two smoking groups (χ² = 4.70, p = 0.195). Among never smokers, stage I disease was most common, affecting 39(42.4%) patients, followed by stage II in 30 (32.6%) patients, stage III in 18 (19.6%) patients, and stage IV in 5(5.4%) patients. A similar pattern was observed among smokers, with stage I disease in 358 (52.7%) patients, stage II in 197 (29.0%) patients, stage III in 105 (15.5%) patients, and stage IV in 19 (2.8%) patients. These results suggest that smoking status was not significantly associated with disease stage at diagnosis in this cohort.

Overall, Table 1 demonstrates that smoking status is significantly associated with sex and lung cancer subtype but not with age at diagnosis or pathologic stage. These differences emphasize the importance of accounting for demographic and tumor characteristics when evaluating smoking-associated DNA methylation patterns in lung cancer.

Table 2 presents the top smoking-associated differentially methylated CpG sites identified from multivariable DNA methylation analysis in lung cancer patients. The listed seven CpG sites were significantly associated with smoking status (FDR < 0.05), with moderate to large effect sizes (|Δβ|: 0.100-0.123). Overall, smoking was associated with a mixed pattern of methylation changes, including four hypomethylated and three hypermethylated loci, indicating bidirectional epigenetic alterations. Collectively, these results demonstrate that smoking status is independently associated with distinct DNA methylation alterations in lung cancer, supporting the role of tobacco exposure in shaping tumor-specific epigenetic profiles.

**Table 2:** Top differentially methylated CpG sites associated with smoking status in TCGA lung cancer cohort Δβ represents the mean difference in methylation beta values between smokers and never smokers, with positive values indicating higher methylation in smokers and negative values indicating lower methylation in smokers. Multivariable linear models were fitted using the limma framework, adjusting for age at diagnosis, sex, tumor stage, and lung cancer subtype. Empirical Bayes moderation was applied to stabilize variance estimates across CpG sites. Statistical significance was assessed using moderated t-statistics. P-values were adjusted for multiple testing using the Benjamini–Hochberg false discovery rate (FDR) method.p < 0.05 was considered statistically significant. Statistically significant p-values are indicated with an asterisk (*). TCGA: The Cancer Genome Atlas

CpG	Δβ	t-statistic	p-value	FDR
cg25636481	-0.102	-4.997	<0.001*	0.007
cg00800229	0.123	4.916	<0.001*	0.008
cg10731960	-0.100	-4.758	<0.001*	0.012
cg01897797	0.103	4.515	<0.001*	0.019
cg24135583	-0.101	-4.359	<0.001*	0.023
cg07558472	0.103	4.181	<0.001*	0.029
cg26090107	0.108	3.803	<0.001*	0.043

**Figure 1:**
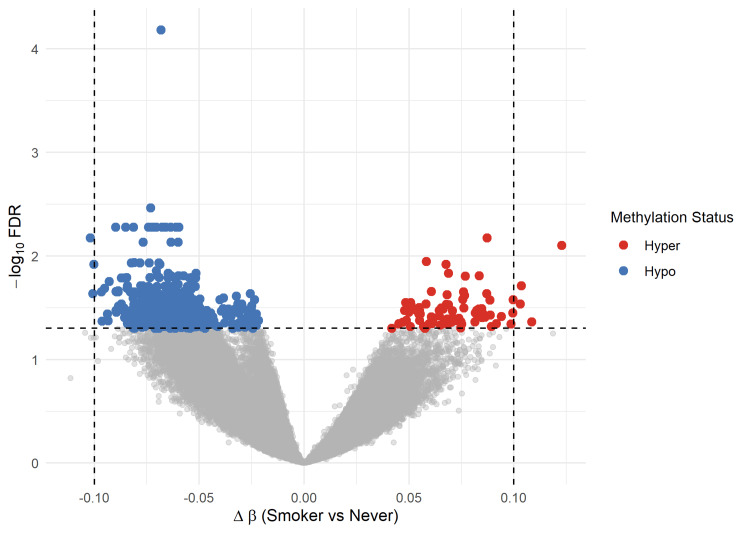
Genome-wide differential DNA methylation associated with smoking status in lung cancer The volcano plot displays CpG sites according to their effect size (Δβ; smokers versus never smokers) on the x-axis and statistical significance (−log10 adjusted p-value) on the y-axis. Each point represents an individual CpG site analyzed using multivariable linear models adjusted for age, sex, tumor stage, and histological subtype. CpG sites reaching false discovery rate (FDR) < 0.05 are highlighted, with positive Δβ values indicating hypermethylation and negative Δβ values indicating hypomethylation in smokers relative to never smokers.

Figure 1 presents a volcano plot summarizing genome-wide differential DNA methylation associated with smoking status among lung cancer patients from the TCGA-LUAD and TCGA-LUSC cohorts. The volcano plot illustrates a distinct set of CpG sites exhibiting significant differential methylation associated with smoking status. Both hypermethylated and hypomethylated loci are observed among smokers, indicating that tobacco exposure is linked to bidirectional epigenetic alterations in lung cancer tissue. The presence of CpG sites with moderate to large effect sizes and strong statistical support highlights the robust association between smoking and tumor-specific DNA methylation changes, independent of key clinical and pathological covariates.

**Figure 2:**
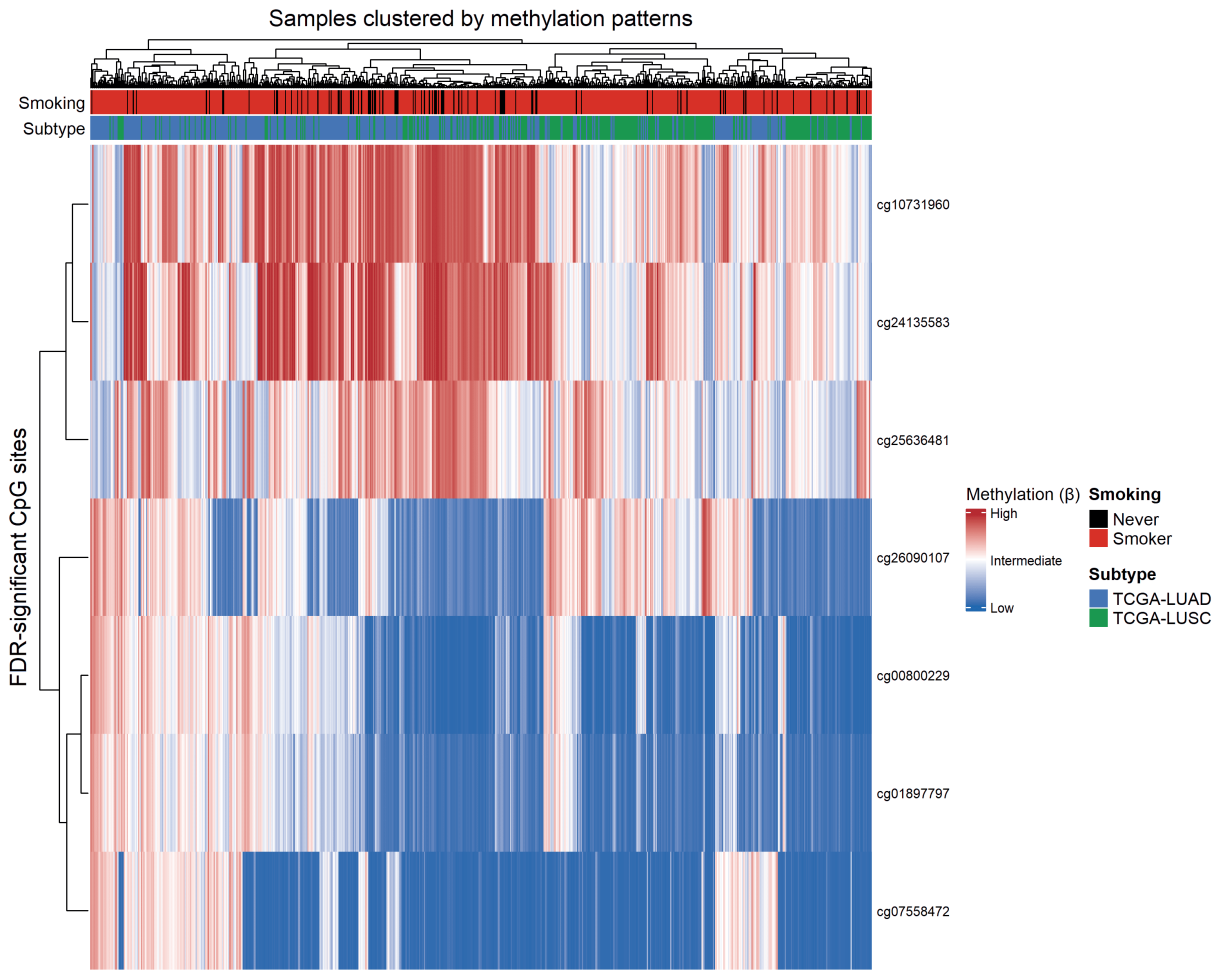
Heatmap of smoking-associated differentially methylated CpG sites in lung cancer The heatmap depicts beta values for the top differentially methylated CpG sites identified at FDR < 0.05. Rows correspond to CpG sites, and columns represent individual tumor samples. Methylation levels are scaled by row to emphasize relative differences across samples. Samples are annotated by smoking status (smokers versus never smokers) and lung cancer subtype (LUAD or LUSC). Hierarchical clustering was applied to both CpG sites and samples using Euclidean distance and complete linkage. LUSC: lung squamous cell carcinoma; LUAD: lung adenocarcinoma; FDR: false discovery rate

Figure 2 presents a heatmap illustrating methylation patterns of the most significant smoking-associated CpG sites across lung cancer samples. The heatmap reveals clear clustering patterns that distinguish smokers from never smokers based on DNA methylation profiles. Several CpG sites show consistent differences in methylation levels according to smoking status, supporting the presence of coordinated epigenetic alterations associated with tobacco exposure. Additionally, subtle subtype-specific clustering patterns are evident, suggesting that smoking-related methylation changes may interact with tumor histology.

Table 3 presents subtype-specific smoking-associated differentially methylated CpG sites identified separately within LUAD and LUSC. Subtype-specific analysis identified eight LUAD-specific and five LUSC-specific CpG sites (FDR < 0.05), demonstrating distinct epigenetic patterns by tumor subtype. LUAD exhibited a mixed pattern of hypermethylation and hypomethylation, whereas LUSC showed a consistent pattern of hypomethylation across all identified loci.

**Table 3:** Subtype-specific differentially methylated CpG sites associated with smoking in LUAD and LUSC Δβ represents the difference in mean methylation beta values between smokers and never smokers within each subtype, with positive values indicating higher methylation in smokers and negative values indicating lower methylation in smokers. False discovery rate (FDR)–adjusted p values were calculated using the Benjamini–Hochberg method. Subtype-specific CpG sites were defined as those achieving statistical significance (FDR < 0.05) in one subtype while not meeting significance criteria in the other subtype. Lung adenocarcinoma (LUAD) and lung squamous cell carcinoma (LUSC) analyses were conducted using multivariable linear models adjusting for age at diagnosis, sex, and tumor stage.

CpG	subtype pattern	Δβ LUAD	FDR LUAD	Δβ LUSC	FDR LUSC
cg22132788	LUAD-specific	0.119	0.002	–	–
cg19089201	LUAD-specific	0.118	0.003	–	–
cg16873863	LUAD-specific	0.109	0.033	–	–
cg12056501	LUAD-specific	-0.111	0.047	–	–
cg26550235	LUAD-specific	0.133	0.047	–	–
cg23179456	LUAD-specific	0.172	0.047	–	–
cg08900158	LUAD-specific	-0.109	0.047	–	–
cg00800229	LUAD-specific	0.139	0.047	–	–
cg05151154	LUSC-specific	–	–	-0.149	0.011
cg08319991	LUSC-specific	–	–	-0.114	0.012
cg13393978	LUSC-specific	–	–	-0.108	0.012
cg15075851	LUSC-specific	–	–	-0.114	0.014
cg25999442	LUSC-specific	–	–	-0.110	0.042

## Discussion

This study aimed to identify smoking-associated differentially methylated regions and evaluate their relevance within a clinically meaningful lung cancer context. Using TCGA methylation profiles from LUAD and LUSC, the findings demonstrate that smoking status is associated with widespread, statistically significant DNA methylation changes that are consistent across subtypes while still reflecting known biological and histological differences.

The differential methylation patterns observed between smokers and never-smokers support the established role of tobacco exposure as a major epigenetic modifier in lung carcinogenesis [10,12]. The volcano plot illustrates numerous CpG sites with significant Δβ values, indicating both hypermethylation and hypomethylation events associated with smoking. These modest but reproducible methylation shifts are characteristic of exposure-related epigenetic alterations and have been shown to exert functional effects on gene regulation when occurring across multiple regulatory regions [13,15]. This study’s results align with previous epigenome-wide studies demonstrating that smoking leaves a persistent methylation imprint in lung tumors beyond genetic mutations alone [5,14].

Clinically, the presence of smoking-associated methylation changes across both LUAD and LUSC highlights the shared upstream impact of tobacco exposure despite divergent genetic drivers. LUSC is strongly linked to smoking and characterized by a high mutational burden resulting from tobacco carcinogens, whereas LUAD more frequently arises in never-smokers and is enriched for targetable alterations such as EGFR mutations and ALK rearrangements [7,9]. The clustering patterns observed in the heatmap indicate that smoking-related epigenetic remodeling operates across histological subtypes but is modulated by tumor lineage and genetic background, reinforcing the concept that genetic and epigenetic mechanisms act synergistically in lung cancer development [8].

From a histopathological perspective, the detection of smoking-associated methylation signatures independent of subtype suggests that epigenetic alterations may precede overt morphological differentiation. This supports the notion that smoking contributes to early molecular priming of lung epithelial cells, influencing tumor phenotype and progression [2]. Importantly, the persistence of these methylation patterns in tumors from former smokers is consistent with epidemiological data showing sustained lung cancer risk after smoking cessation, underscoring the long-term biological impact of tobacco exposure [3].

These findings also align with the concept of field cancerization in the lung. Chronic smoking induces widespread epigenetic alterations across the respiratory epithelium, creating a molecularly altered field that increases susceptibility to malignant transformation [10,12]. Smoking-related methylation changes have been identified in non-tumorous lung tissue, sputum, and blood, suggesting that these alterations arise early and extend beyond the tumor itself [18]. The coherent methylation patterns detected in this study provide tumor-level evidence supporting this field effect and help explain the multifocal nature of smoking-related lung cancers.

Therapeutically, the identification of smoking-associated methylation changes has important implications. Unlike genetic mutations, DNA methylation alterations are potentially reversible, making them attractive targets for epigenetic therapies such as DNA methyltransferase inhibitors [17]. Furthermore, smoking-related epigenetic regulation of immune and inflammatory pathways may influence tumor immune landscapes and responses to immunotherapy, which are known to differ between smoking-related and non-smoking-related tumors [8]. Integrating methylation profiling into clinical decision-making may therefore enhance patient stratification and treatment optimization.

Finally, the stability and detectability of DNA methylation marks highlight their potential as biomarkers for lung cancer risk assessment and early detection. Smoking-associated methylation signatures identified in tumor tissue overlap with markers detectable in minimally invasive samples, supporting their translational relevance for screening and disease monitoring in high-risk populations [13,18]. Alam et al. reported smoking‑related changes in oral microbial and tissue contexts, supporting exploration of whether tumor methylation markers overlap with signals detectable in oral or sputum samples [19].

Strengths and limitations

This study leveraged a large, well-annotated TCGA cohort (n = 771), enabling robust, subtype-specific (LUAD and LUSC) analysis of smoking-associated DNA methylation patterns within a clinically relevant framework. The use of multivariable linear modeling with empirical Bayes moderation and false discovery rate correction strengthened the stability and reliability of the findings.

However, several limitations should be considered. First, the cross-sectional design limits causal inference. Second, smoking exposure was modeled as a binary variable (smoker vs. never smoker) due to limitations in TCGA clinical data, where detailed quantitative measures such as pack-years are not consistently available. While this approach enabled inclusion of the full cohort, it may not fully capture dose-response relationships. Third, the analysis was restricted to tumor samples without inclusion of matched normal lung tissue, limiting the ability to distinguish cancer-specific methylation changes from baseline epigenetic variation and constraining assessment of early field effects.

Additionally, the relatively smaller number of never smokers (n = 92) compared to smokers may introduce imbalance and affect statistical precision in subgroup analyses. Furthermore, although standard preprocessing and filtering steps were applied, detailed adjustment for potential batch effects, normalization differences, and probe-specific biases was limited. Importantly, the lack of adjustment for tumor purity and cell-type heterogeneity represents a key limitation, as observed methylation differences may partially reflect underlying cellular composition rather than smoking exposure alone.

Finally, the absence of multi-omics integration, as this study focused solely on DNA methylation profiles. Integrating methylation data with gene expression or other molecular layers was beyond the scope of the current analysis but would strengthen the functional interpretation of identified CpG sites and help distinguish biologically relevant signals from potential technical or cellular composition effects. Prior studies have shown that smoking-associated CpG sites in lung tissue often correlate with gene expression changes across lung cancer subtypes, supporting the importance of such integrative approaches. Future work should incorporate multi-omics analyses to validate and extend the functional relevance of these findings.

Future studies integrating multi-omics data, incorporating normal comparator samples, accounting for tumor purity and cellular composition, and utilizing more detailed exposure metrics are warranted to better elucidate the biological and clinical implications of smoking-associated epigenetic changes in lung cancer.

## Conclusions

This study shows that smoking is strongly associated with distinct DNA methylation changes across lung cancer subtypes using TCGA data. Smokers exhibited reproducible epigenetic alterations that differed between LUAD and LUSC, reflecting known biological and clinical differences. These findings support the role of smoking in shaping the lung cancer epigenome alongside genetic drivers. The persistence and subtype specificity of these methylation patterns highlight their potential value as biomarkers for risk assessment, disease characterization, and future epigenetic therapeutic strategies.
